# Island Cotton Enhanced Disease Susceptibility 1 Gene Encoding a Lipase-Like Protein Plays a Crucial Role in Response to *Verticillium dahliae* by Regulating the SA Level and H_2_O_2_ Accumulation

**DOI:** 10.3389/fpls.2016.01830

**Published:** 2016-12-15

**Authors:** Zhang Yan, Wang Xingfen, Rong Wei, Yang Jun, Ma Zhiying

**Affiliations:** Department of Agronomy, North China Key Laboratory for Germplasm Resources of Education Ministry, Hebei Agricultural UniversityBaoding, China

**Keywords:** cotton (*Gossypium barbadense*), *EDS1*, *Verticillium* wilt resistant, transgenic *Arabidopsis*, virus-induced gene silence, SA level, H_2_O_2_ accumulation

## Abstract

Cotton is one of the most economically important crops, but most cultivated varieties lack adequate innate immunity or resistance to *Verticillium* wilt. This results in serious losses to both yield and fiber quality. To identify the genetic resources for innate immunity and understand the pathways for pathogen defenses in this crop, here we focus on orthologs of the central *Arabidopsis thaliana* defense regulator *Enhanced Disease Susceptibility* 1 (*EDS1*). The full-length cDNA of *GbEDS1* was obtained by screening the full-length cDNA library of *Gossypium barbadense* combining with RACE strategy. Its open reading frame is 1848 bp long, encoding 615 amino acid residues. Sequence analysis showed that GbEDS1 contains a conserved N-terminal lipase domain and an EDS1-specific KNEDT motif. Expression profiling indicated that the gene is induced by *Verticillium dahliae* as well as salicylic acid (SA) treatment. Subcellular localization assays revealed that GbEDS1 is located in the cell cytoplasm and nucleus. Overexpression of *GbEDS1* in *Arabidopsis* dramatically up-regulated SA and H_2_O_2_ production, resulting in enhanced disease resistance to *V. dahliae*. Silencing of *GbEDS1* in *G. barbadense* significantly decreased SA and H_2_O_2_ accumulation, leading to the cotton more susceptibility. Moreover, combining the gene expression results from transgenic *Arabidopsis* and silenced-GbEDS1 cotton, it indicated that *GbEDS1* could activate *GbNDR1* and *GbBAK1* expression. These findings not only broaden our knowledge about the biological role of *GbEDS1*, but also provide new insights into the defense mechanisms of *GbEDS1* against *V. dahliae* in cotton.

## Introduction

Cotton (*Gossypium* sp.) is one of the most economically valuable crops globally. Because its products are used as textile fibers, feed stock, foodstuff, oil, and biofuels. However, its yields have been severely reduced due to increasingly unfavorable environmental conditions, including those associated with biotic and abiotic stresses. Among these, *Verticillium* wilt, induced by *Verticillium dahliae* Kleb., is one of the most destructive diseases of *G*. *hirsutum* L., and has been detected in most areas where that species is cultivated. Losses to annual yields have risen to more than 1.5 million bales worldwide (Cai et al., 2009).

This soil-borne pathogen penetrates the roots and systemically infects the plant through the xylem (Daayf et al., 1995; Klosterman et al., 2009). Affected cotton plants usually exhibit symptoms of chlorosis or necrosis in the leaves, discoloration of the stem vascular bundles, a decrease in photosynthesis and an increase in respiration, and significant declines in plant biomass and yield (Sink and Grey, 1999). So far, developing resistant cultivars has been regarded as the most effective strategy in combating this stubborn disease. Although the introduction of genetic resistance is considered the best and most sustainable management approach, a lack of information about the molecular mechanisms for cotton resistance against *V. dahliae* has slowed progress in those research efforts.

Complex molecular mechanisms protect plants from the effects of insects and pathogens. These include constitutive physical and chemical barriers that hinder pathogen entry and infection as well as a powerful defense system that can induce the expression of various stress-related genes (Creelman and Mullet, 1997; Reymond and Farmer, 1998; Glazebrook, 2005; Jones and Dangl, 2006; Howe and Jander, 2008; Fu et al., 2012). The core genetic components that regulate upstream events in the biosynthesis of salicylic acid (SA) include *EDS1*, *PAD4*, and *SAG101* (Feys et al., 2001; Wiermer et al., 2005; Pieterse et al., 2009; Rietz et al., 2011). In *Arabidopsis thaliana*, *EDS1* is a key positive regulator of basal resistance to biotrophic and hemi-biotrophic pathogens and it participates in effector-triggered immunity that is mediated by a subset of resistance genes (Aarts et al., 1998; Feys et al., 2001; Wiermer et al., 2005; Wirthmueller et al., 2007). *EDS1* could control the production of SA required for basal defense and systemic resistance against virulent pathogens (Attaran et al., 2009; Vlot et al., 2009) and is essential for ETI (Feys et al., 2001, 2005; Wirthmueller et al., 2007). In SA signal pathway, *EDS1* together with *PHYTOALEXIN DEFICIENT4* (*PAD4*) also needed for many SA-independent gene expression changes in response to infection (Falk et al., 1999; Jirage et al., 1999; Wiermer et al., 2005; Wang L. et al., 2008). *EDS1/PAD4* could indirectly up-regulate the expression of isochorismate synthase (SALICYLIC ACID INDUCTIOND EFICIIENT2/ISOCH OR ISMATE SYNTHASE1, SID2/ICS1), an enzyme required for SA synthesis (Wildermuth et al., 2001). Furthermore, the expression of *EDS1* could be regulated by calmodulin (*CAMTA3*), which interacts with the promoter of *EDS1* and represses its expression. So *EDS1* plays critical role in Ca^2+^/calmodulin regulates salicylic-acid-mediated plant immunity (Du et al., 2009; Truman et al., 2013; Zhang et al., 2014). In addition, *EDS1* plays a crucial role in transducing redox signals in response to biotic and abiotic stresses (Mateo et al., 2004; Straus et al., 2010). Those reactions include the production of SA, reactive oxygen species (Rusterucci et al., 2001), and secondary metabolites, as well as the expression of both SA-dependent and -independent defense genes (Bartsch et al., 2006).

All of these reports suggest that *EDS1* plays crucial roles in biotic/abiotic stresses (Attaran et al., 2009; Du et al., 2009; Vlot et al., 2009; Truman et al., 2013; Zhang et al., 2014). Most previous studies of *EDS1* gene have emphasized model species such as *Arabidopsis* and tomato (Rusterucci et al., 2001; Liu et al., 2002; Hu et al., 2005; Rietz et al., 2011). As one of the most important fiber and oil crops, cotton experiences severely impaired growth and yields when exposed to *V. dahliae*. Therefore, to improve our understanding of the resistance/defense mechanism and identify more resistance genes, we constructed a SSH library and a full- length cDNA library from the tolerance cultival cv. Jimian20 and resistant *G. barbadense* cv. Pima90-53, respectively (Wang X.F. et al., 2008; Zhang et al., 2013). From both cDNA library, we detected *EDS1* gene and found it involved in cotton defense against *V. dahliae*. However, cotton *EDS1* gene has not been studied deeply except Su’ report (2014). In this study, we overexpressed *GbEDS1* in *Arabidopsis* plants and then investigated the effect on *V. dahliae* invasion and the molecular mechanism of *GbEDS1* in resistance to *V. dahliae*.

## Materials and Methods

### Plant Growth and Seedling Treatments

Seeds of *Gossypium barbadense* cv. Pima90–53 (resistant) were germinated in Hoagland solution-saturated vermiculite in a controlled environment chamber [28/25°C (day/night), 16-h photoperiod, and 80% relative humidity]. Plants of the wild-type (WT) *A. thaliana* Columbia ecotype and transgenic lines (see below) were grown at 22°C, under long-day conditions (16 h light/8 h dark) (Haffner et al., 2010) in the greenhouse.

The highly aggressive defoliating *V. dahliae* fungus strain, isolated from field-grown upland cotton plants that showed typical symptoms of infection, was cultured on a potato-dextrose agar medium for 7 days at 25°C. Colonies were then incubated for 5 days in Czapek’s medium [NaNO_3_ (0.3% w/v), MgSO_4_ (0.1% w/v), KH_2_PO_4_ (0.1% w/v), FeSO_4_ (0.0002% w/v), KCl (0.1% w/v), and sucrose (3% w/v); pH 6.0] at 25°C. Prior to inoculation treatments, the concentration of spores was adjusted to approximately 10^7^ conidia per mL with deionized water. We used a GFP-tagged *V. dahliae* strain (Vd-gfp77), provided by Professor Dai XF (Chinese Academy of Agricultural Sciences), to observe the process of colonization during the experimental period. Its pathogenicity on cotton is related to that of the WT isolate Vd991 (Xu et al., 2013). When the cotton seedlings were 10 days old, they were treated with 10 mM methyl salicylate (MeSA) (Wang et al., 2007), which is converted to SA in the leaf tissue (Shulaev et al., 1997). For pathogen treatment, 10-day-old seedlings were uprooted gently and their roots rinsed in distilled water. The roots were then dipped for 3 min in the conidia suspension. Control plants were treated similarly with distilled water (Zhang et al., 2011). The root tissues were harvested separately from all the treatments after 0, 12, 24, 48, 72, and 96 h and were frozen in liquid nitrogen immediately.

### RNA Preparation and Gene Cloning

Total RNA was extracted from cotton roots with a PlantRNA Kit (Tiangen, China) according to the manufacturer’s protocol. First-strand cDNA was synthesized from 5 μg of total RNA with a StrataScript Kit (Stratagene USA). Based on the candidate sequence of *GbEDS1* from the full-length cDNA library, we designed a pair of primers (GbEDS1–F1/GbEDS1–R1) according to the lateral flanking sequence of the open reading frame (ORF) of the candidate sequence. The primers are listed in Supplementary Table S1.

### Bioinformatics Analysis

Nucleotide and deduced amino acid sequences were investigated through an NCBI/GenBank/Blast. Sequences were aligned and compared with those of other species via DNAMAN, and a phylogenetic tree was constructed for *GbEDS1* and similar genes from other plants. The signal sequences were predicted with SignalP^1^. Functional regions and activity sites were identified from the PROSITE^2^ and SMART motif search programs^3^. Sub-cellular localization was predicted by Psort (Collings, 2013).

### Gene Expression Analysis

Total RNA was extracted from cotton as previously described (Zhang et al., 2011). First-strand cDNA was synthesized from 1 μg of total RNA using the iScript^TM^ cDNA synthesis Kit (Bio-Rad, USA) system. For RT-PCR, gene-specific primers were used to analyze the change in *GbEDS1* expression between mock- and *V*. *dahliae*-inoculated plants. *GhUBQ14* (GenBank: DW505546) from cotton was used as the reference gene to normalize the total amount of cDNA in each reaction (Artico et al., 2010). All qPCRs were performed according to the guidelines of the Minimum Information for Publication of Quantitative Real Time PCR Experiments (Bustin et al., 2009). Diluted cDNA was used with SYBR Green on an ABI 7500 Real Time PCR system (Applied Biosystems, USA). Three biologically independent experiments were run for this analysis. Relative fold-changes were calculated per the 2^-ΔΔCt^ method, as described by Livak and Schmittgen (2001). All primers are shown in Supplementary Table S1.

### Subcellular Localization of GbEDS1::GFP Fusion Proteins

To find the cellular localization of GbEDS1, we amplified the coding region of *GbEDS1* with GbEDS1–F1/GbEDS1–R2 primers and cloned it into the pCamE-GFP vector to generate a GbEDS1–GFP in-frame fusion with green fluorescence protein (GFP). The plasmid was introduced into onion epidermal cells by particle bombardment, as previously described (Collings, 2013). Afterward, the tissues were incubated for 24 h on an MS agar medium under darkness at 22°C before being examined with a fluorescence microscope.

### Generation of Transgenic *Arabidopsis* Plants

The ORF of *GbEDS1* was amplified with primers GbEDS1-F1 and GbEDS1-R1 (Supplementary Table S1), and then inserted into binary vector pBI121 under the control of the CaMV 35S promoter via *Xba*I and *Sac*I sites. The recombinant construct vector was introduced into *Agrobacterium tumefaciens* (strain GV3101), and then transferred into eds1 mutant plants of *Arabidopsis* (Columbia background) by floral-dipping (Clough and Bent, 1998). The transformants were first screened on kanamycin (100 mg L^-1^) plates and then verified by RT-PCR using primers GbEDS1-F4/GbEDS1-R4 (Supplementary Table S1). Transgenic T_3_ lines were used for further experiments.

### Assay for Disease Resistance

*Verticillium* wilt resistance was assessed in seedlings that had been gently inoculated with *V. dahliae* as we previously described (Zhang et al., 2011). All plants were irrigated with Hoagland’s nutrient solution. Disease development was monitored for up to 28 days post-inoculation (dpi). The degree of wilt resistance was evaluated along a scale of disease grades from 0 to 4 (Ma et al., 2000). The disease index was calculated according to the following formula: DI = [(Σ disease grades × number of infected plants) / (total number of checked plants × 4)] × 100 (Zhang et al., 2011). Values obtained for DI represented the status of infection for a population, with lower DI values indicating that the plants were more resistant.

We also quantified the *V. dahliae* biomass produced in transgenic plants (Jun et al., 2015). After visible symptoms were observed at 14 dpi, all aboveground tissues from each *Arabidopsis* genotype were harvested and flash-frozen in liquid nitrogen. The samples were ground to powder and approximately 100 mg of each was used for DNA isolation. Three-step Q-PCR was performed on a LightCycler 1.5 Instrument (Roche, Germany) using a SYBR Premix Ex Taq^TM^ II kit (TaKaRa). For measuring the *V. dahliae* biomass, the internal transcribed spacer (ITS) region of the ribosomal DNA was targeted to generate a 200-bp amplicon by the fungus-specific primers ITS1-F and ST-VE1-R (Gardes and Bruns, 1993; Lievens et al., 2006). The large subunit of the RuBisCo gene from *Arabidopsis* was used for sample equilibration. The average fungal biomass was determined from at least three *Verticillium*-inoculated plants per genotype, and quantification was conducted as described by Ellendorff et al. (2009).

### Virus-Induced Gene Silencing (VIGS) in Cotton

To investigate the role of *EDS1* in *Verticillium* resistance, we used virus-induced gene silencing (VIGS). The TRV vectors and *Agrobacterium tumefaciens* for VIGS were prepared according to the method of Gao et al. (2011). Insert fragments to generate *TRV::GbEDS1* and the positive control *TRV::GbCLA1* were amplified from *G. barbadense* cv. Pima90-53. Primers to produce the TRV vectors are listed in Supplementary Table S1. The TRV2 plasmid and *GbEDS1* fragment were digested with *Xba*I and SacI, then ligated using T4 DNA ligase (Takara, Japan), and transformed into *A. tumefaciens* GV3101 by electroporation. The *TRV::GbCLA1* was obtained using the same method for TRV::GbEDS1. Subsequently, TRV vectors were agro-infiltrated (Gao et al., 2011) into the cotyledons of 7-day-old seedlings of *G. barbadense* cv. Pima90-53. The seedlings were then grown at 25°C under a 16-h photoperiod in a controlled environment chamber. A distinct bleaching phenotype for the leaves was observed at 10 days after infiltration in *TRV::GbCLA1* plants. Therefore, inoculation with *V. dahliae* was performed at that time point. Two weeks after inoculation, wilt resistance began assessed. The percentage of diseased plants and a disease index (see below) were determined from about 30 seedlings per treatment and the assessment was repeated at least three times.

### Expression Analysis of Defense-Related Genes in Transgenic *Arabidopsis* Plants

To study whether *GbEDS1* overexpression in *Arabidopsis* influences SA signal transduction and the expression of defense-related genes, we monitored the expression of SA pathway genes (*EDS1*, *NPR1*, *NDR1*) (Shah, 2009; Vlot et al., 2009; Fu and Dong, 2013), a co-receptor and signaling regulator of different pattern recognition receptors—*BRI1-associated kinase 1* (*BAK1*) (Gao et al., 2013), and defense marker genes (*PR1*, *PR5*) in transgenic *Arabidopsis*. For the 20-day transgenic *Arabidopsis* seedlings were removed gently from vermiculite and their roots were washed in sterile water. Then root part was dipped in the fresh spore suspension (about 10^7^ conidia/mL). After inoculation, the seedlings were re-planted in the pot containing new vermiculite and were irrigated with Hoagland’s nutrient solution. Leaf tissues were sampled separately from transgenic and wild type *Arabidopsis* after 0, 6, 12, 24 hpi and were frozen in liquid nitrogen. The specificity of the primers in real-time PCR was tested by PCR amplification and sequencing. All PCR procedures were repeated three times and the data were normalized to reference genes according to the 2^-ΔΔCT^ method (Livak and Schmittgen, 2001).

### Measurements of Total SA

In *Arabidopsis*, *EDS1* was considered to be one of essential mediators in SA signaling (Falk et al., 1999; Jirage et al., 1999; Wiermer et al., 2005). SA and *AtEDS1* were considered to function in a positive feedback regulatory loop (Bartsch et al., 2006). Thus, we want to know whether *GbEDS1* could regulate SA level changes. Extraction and measurements of endogenous SA from seedlings were performed as previously described (Nandi et al., 2004). For each sample, 200 mg of leaves was ground and extracted once with 3 mL of 90% methanol and once with 3 mL of 100% methanol. The combined extracts were dried under N_2_ gas and suspended in 2.5 mL of 5% trichloroacetic acid. Afterward, the samples were acid-hydrolyzed by adding 200 mL of HCl and incubating in a boiling water bath for 30 min. The SA was extracted with 5 mL of a mixture containing cyclohexane:ethylacetate:isopropanol (50:50:1). Samples were dried under N_2_ gas and dissolved in 0.5 mL of the mobile phase (69:27:4 mix of water:methanol:glacial acetic acid) (Dadgar et al., 1985). They were then passed through a 0.22-mm filter, and 20 to 100 μL was used for HPLC. Concentrations of SA were determined by comparing our samples with available standards.

### Determination of H_2_O_2_ Content

H_2_O_2_ accumulation has been considered to be an important parameter in biotic stresses because it usually regarded as an immunity-associated molecule (Camejo et al., 2016). To further investigation the function of *GbEDS1* in defense against *V. dahliae*, we monitored the levels of H_2_O_2_ in transgenic line and GbEDS1-silenced plants. The seedlings leave inoculated with *V. dahliae* were sampled. Each sample was homogenized in pre-cooled phosphate-buffered saline (PBS), using 1 mL of buffer per 0.1 g of fresh tissue. The homogenate was centrifuged at 10,000 × *g* for 10 min at 4°C. Freshly isolated supernatant fractions were used immediately for measuring H_2_O_2_ with commercial kits (Jiancheng Biotech Inc., Nanjing, China). Adduct formation was measured spectrophotometrically at 405 nm using Thermo Scientific Multiskan FC (Shanghai, China) in strict accordance with the manufacturer’s instructions. Protein contents were determined with an Enhanced BCA Protein Assay Kit (Beyotime, Shanghai, China).

### Analyses of SOD and POD Activities

The 4-week old *Arabidopsis* grown in pots were inoculated with *V. dahliae* according to the method of Zhang et al. (2011). Then the leaves from transgenic and mock plants were sampled at 6, 12, 24, 36, 48 hpi, respectively. The activities of superoxide dismutase (SOD) and peroxidase (POD) in each sample were measured using commercial kits (Jiancheng Biotech Inc., Nanjing, China) according to the manufacturer’s instructions. Protein contents were determined with an Enhanced BCA Protein Assay Kit (Beyotime, Shanghai, China).

### Statistical Analysis

The experiments were repeated three times. All data were presented as means ± SE. Differences among treatments were evaluated with two-tailed unpaired Student’s *t*-tests, a value of *P* < 0.05 or *P* < 0.01 was considered to be statistically significant.

## Results

### Cloning and Characterization of the Full-Length *GbEDS1* cDNA

**FIGURE 1:**
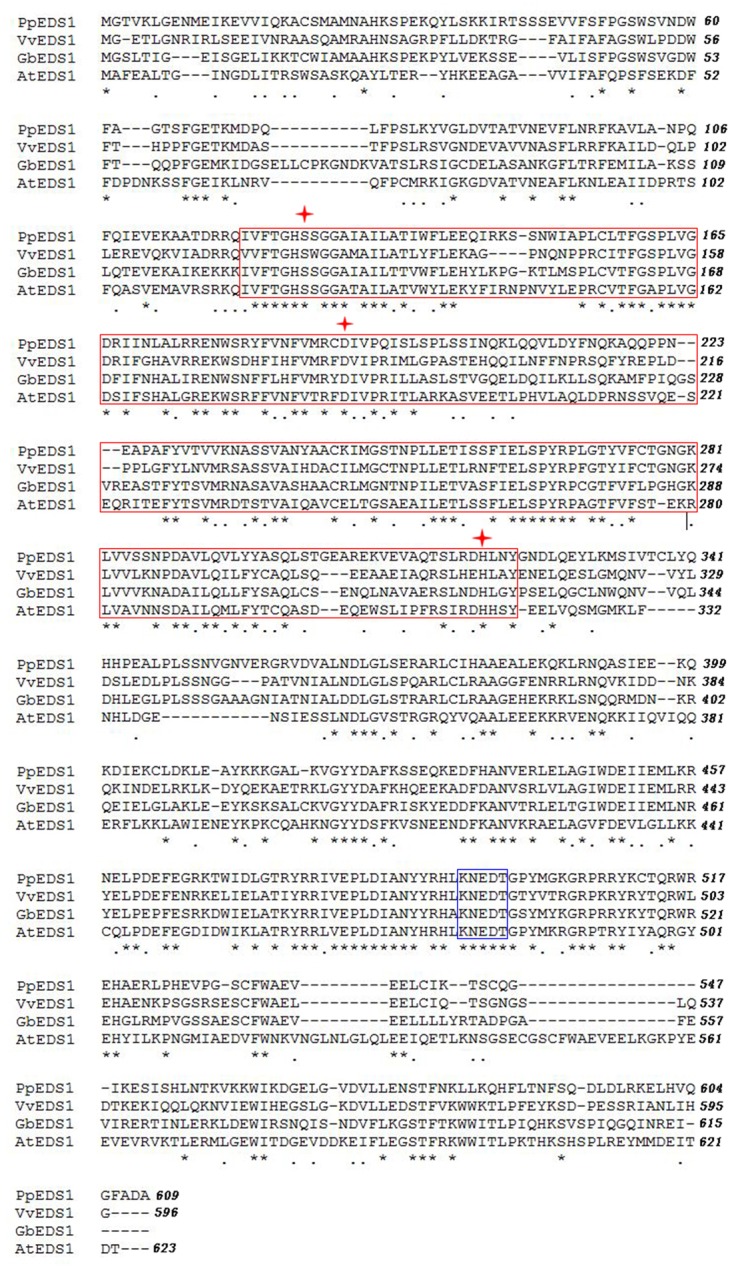
**Amino acid sequence alignment of EDS1 proteins from different plant species evaluated via ClustalW2.** Putative lipase domain and EDS1-specific KNEDT motif are marked with red box and blue box, respectively. Serine (S), aspartate (D), and histidine (H) residues of putative lipase catalytic triad are indicated by asterisks. Pp, *Populus poplar*; Vv, *Vitis vinifera*; Gb, *Gossypium barbadense*; and At, *Arabidopsis thaliana*.

**FIGURE 2:**
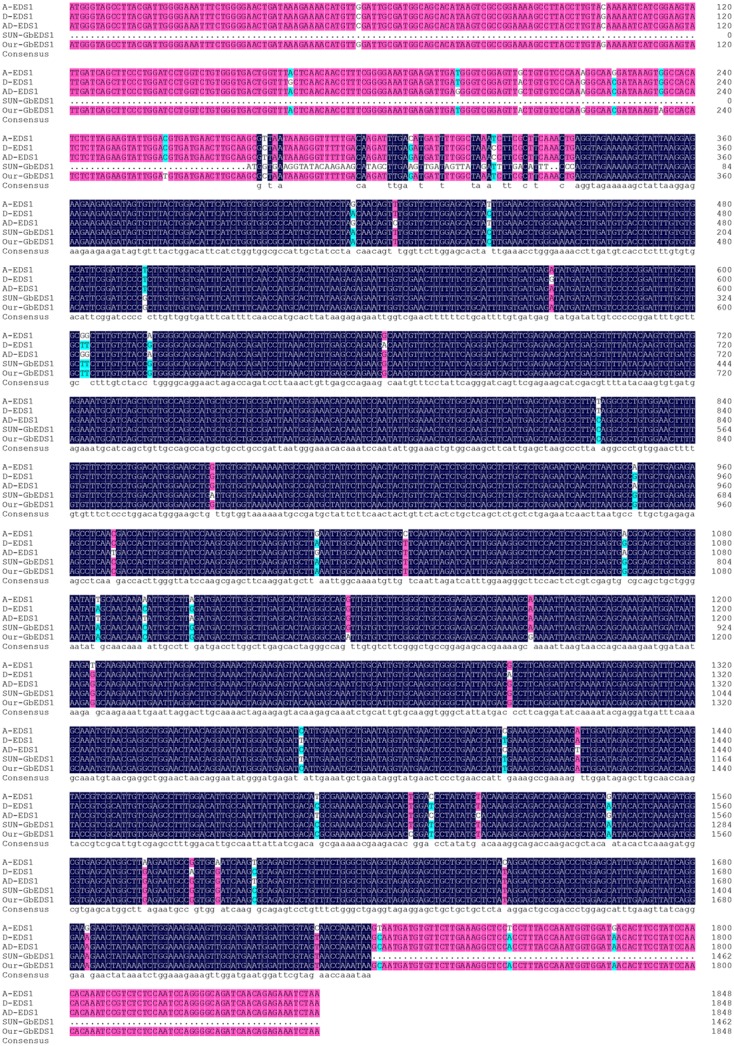
**Alignment of the sequences of *GbEDS1* with its homologous genes in cotton.** Comparison of the sequence of *EDS1* from *G. barbadense*, *G. hirsutum*, *G. arboreum*, and *G. raimondii*.

Using the full-length cDNA library previously constructed from root samples of *G. barbadense* cv. Pima90-53 plants that had been inoculated by *V. dahliae* (Zhang et al., 2013), we obtained a 1706-bp fragment with a poly-A tail. Here, we employed a RACE strategy with GSPs and UPM to amplify the 5′-ends. The complete cDNA of *GbEDS1* was assembled and firstly submitted to GenBank (Accession Number EU855795). The full length cDNA of *GbEDS1* is 2258 bp, containing an ORF of 1848 bp, as well as a 201-bp 5′-UTR and a 206-bp 3′-UTR. ExPASy-Prosite analysis indicated that *GbEDS1* has a conservative lipase-serine active-site signature “IVFTGHSSGG” (Supplementary Figure S1). In addition, this gene displays conserved serine (S), aspartic acid (D), and histidine (H) residues that form a putative hydrolase catalytic triad within its N-terminal lipase-like region (**Figure 1**) (Falk et al., 1999). In that putative lipase domain, the C-terminal region contains the specific motif KNEDT, which is highly conserved among *EDS1* sequences from *Populus poplar* (*Pp*), *Vitis vinifera* (*Vv*), and *A*. *thaliana* (*At*) (**Figure 1**; **Table 1**), suggesting that our *GbEDS1* is an ortholog of *AtEDS1* and *VvEDS1*. Furthermore, GbEDS1-homologous sequences from three other *Gossypium* reference genome and previously reported cotton *EDS1* gene from Su’ research were analyzed by BLAST. Sequences blast showed that there was only one copy of *EDS1* gene in cotton genome. In particular, our *GbEDS1* was more closely related to *GrEDS1* (99% identity) than to *GhEDS1* (98% identity) or *GaEDS1* (97% identity) (**Figure 2**; Supplementary Figure S2). All above results indicated that *EDS1* gene was very conservative across different cotton species. However, why the *EDS1* gene from Su’ sequence sharing low identity with *GrEDS1*, *GhEDS1*, and *GaEDS1* were 75.4, 74.4, and 74.6% is still unclear (Su et al., 2014).

**Table 1:** Sequence homology analysis of GbEDS1 with five other EDS1 proteins, based on their complete ORF amino acid sequences.

Protein^a^	GbEDSl	PpEDSl	GmEDSl	S1EDS1	AtEDSl	VvEDSl
GbEDSl	100%					
PpEDSl	64.0%	100%				
GmEDSl	58.8%	61.8%	100%			
S1EDS1	60.9%	63.5%	60.9%	100%		
AtEDSl	54.4%	54.3%	53.7%	53.2%	100%	
VvEDSl	62.2%	65.6%	62.1%	65.1%	53.5%	100%

^a^*Genebank Accession Numbers for EDS1 proteins: GbEDS1, AY262015; PpEDS1, XM-002322106; GmEDS1, FJ517562; SlEDS1, ay796114; AtEDS1, NM-114678; VvEDS1, EF551159; GrEDS1, scaffold251:2115072:2118380; GaEDS1, Chr6:9856127:9859250; GhEDS1, scaffold821.1:539426:542533.*

### Expression of *GbEDS1* was Induced by *V. dahliae* and SA Treatment

**FIGURE 3:**
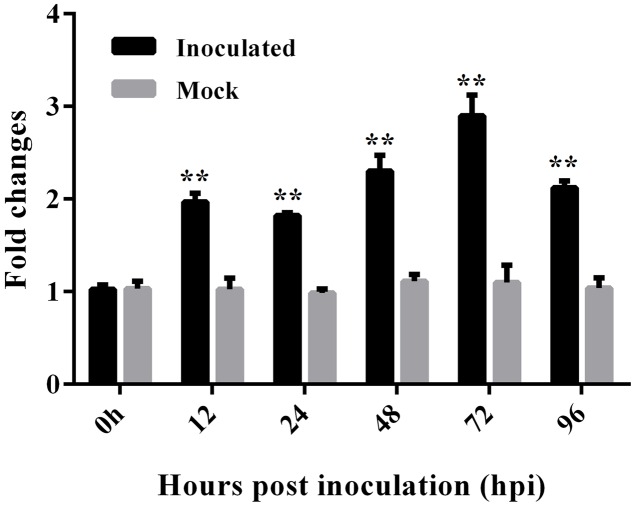
**Expression analysis of *GbEDS1* after induction by *V. dahliae* in root tissues of Pima90-53.** Real-time PCR for time-course tests (12, 24, 48, 72, and 96 hpi) of *GbEDS1* expression in response to *V*. *dahliae*. Bars represent levels of *GbEDS1* transcripts relative to those of cotton *GhUBQ14* (for normalization). Seedlings inoculated with water-only served as control. Data are means ± SD of values obtained from triplicate experiments. Asterisks indicate statistical significance (^∗∗^*P* < 0.01, Student’s *t*-test) in comparison with mock control.

To test the effect of *V. dahliae* infection on the expression of *GbEDS1*, we extracted RNA from roots that were harvested at various time points following inoculation. RT-PCR analysis demonstrated that, compared with the uninfected control, *GbEDS1* was obviously up-regulated in the infected tissues, with transcript levels being highest between 12 and 96 hpi and peaking at 72 hpi (**Figure 3**). This result was evidence that *GbEDS1* gene is responsive to *V. dahliae* infection at the transcriptional level, and implied that it might be involved in defense against *V. dahliae* in *G. barbadense*.

Because *EDS1* and SA are in a positive feedback loop in *Arabidopsis*, so we measured transcript level in *G. barbadense* leaves in response to SA. At 24 h after MeSA treatment, levels of *GbEDS1* transcripts were significantly higher (*P* < 0.01) than the mock treatment (Supplementary Figure S3), suggesting that *GbEDS1* is SA-responsive in *G. barbadense*.

### Subcellular Localization of GbEDS1

**FIGURE 4:**
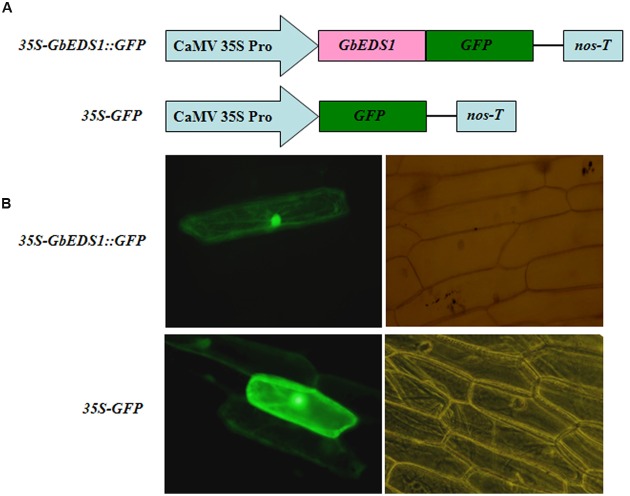
**Subcellular localization of GbEDS1 in onion epidermal cells. (A)**, Schematic diagram of 35S–GbEDS1::GFP and 35S–GFP fusion construct. **(B)**, Transient expression of 35S–GbEDS1::GFP and 35S–GFP. Fluorescence images and bright-field images were presented.

To determine the cellular localization of GbEDS1, we fused its full-length cDNA, except for the stop codon, in-frame with the GFP-coding sequence to yield a GbEDS1–GFP construct under the control of the CaMV 35S promoter (**Figure 4A**). After it was introduced into onion epidermal cells, the GFP signal was detected by fluorescence microscopy. The 35SGbEDS1::GFP construct was localized to the cytoplasm and the nucleus (**Figure 4B**). This was consistent with the targeting of *AtEDS1* in plant cells (Zhu et al., 2011). By contrast, the 35S–GFP control construct showed GFP signals throughout the entire cell (**Figure 4B**).

### Overexpression of *GbEDS1* in *Arabidopsis* Showed Enhanced Defense Response to *V. dahliae*

**FIGURE 5:**
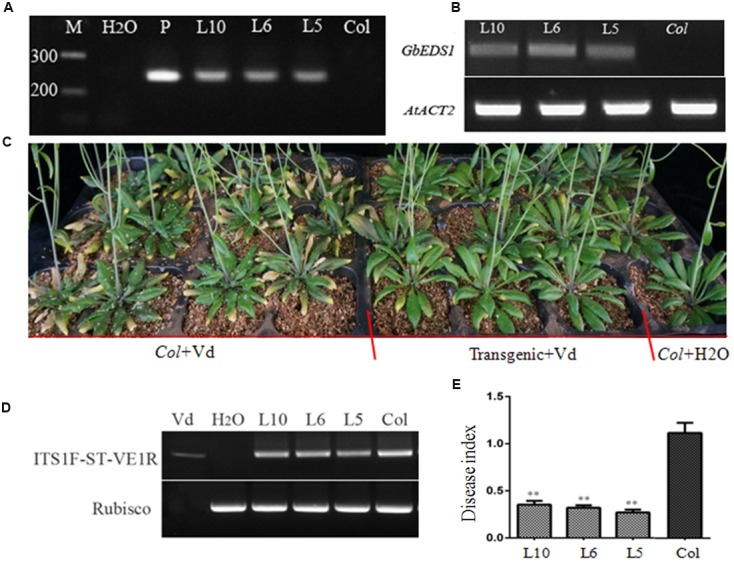
**Overexpression of *GbEDS1* in *Arabidopsis* improved the resistance to *V. dahliae*. (A)** Direct-PCR analysis of genomic DNA from transgenic lines. **(B)** RT-PCR analysis the expression of *GbEDS1* gene in transgenic lines and wilt type plants. M: Marker, H_2_O represented negative control in the PCR system. **(C)** Four-week-old plants were inoculated via root-dipping with 5 × 10^7^ conidia mL^-1^. Similar results were seen from all transgenic lines. Transgenic and wild type plants showed obvious differences in resistance, transgenic plants showed more resistant than wild type plants. **(D)** Detection the biomass of the *V. dahliae* in the transgenic plant with RT-PCR by comparing *V. dahliae* internal transcribed spacer (ITS) transcript levels (as a measure for fungal biomass) relative to *Arabidopsis* Rubisco transcript levels (for equilibration) at 14 days post-inoculation. **(E)** Detection the biomass of the *V. dahliae* in the transgenic plant with Quantitative real-time PCR. The relative average fungal biomass is shown with standard errors. Asterisks indicate significant differences when compared with colonization of the *eds1* mutant. The average fungal biomass was determined using at least three inoculated plants for each genotype. (^∗∗^*P* < 0.01).

Ecotype ‘Col-0’ *Arabidopsis* plants were transformed using *Agrobacterium tumefaciens* to generate transgenic lines overexpressing *GbEDS1* driven by the CaMV35S promoter. Of the 13 independent T_3_ transgenic lines obtained, three (L5, L6, and L10) were selected via genome PCR analysis of the *GbEDS1* fragment (**Figure 5A**). RT-PCR analysis further confirmed that *GbEDS1* was successfully expressed in the transgenic plants (**Figure 5B**). When inoculated with *V. dahliae*, typical symptoms of vascular disease became apparent in the infected wild-type Col-0 plants but much less pronounced in GbEDS1-overexpressing plants at 20 dpi (**Figure 5C**). Moreover, the relative disease index values for the wilt-type plants were 50.0, but for L5, L6, and L10 transgenic lines were 32.8, 31.6, and 34.0, respectively (**Table 2**), indicating that all three transformed lines demonstrated improved wilt resistance. Further evidence that the transgenic lines improved plant resistance were found with regard to the fungal biomass, with significantly less *V. dahliae* biomass was detected in transgenic lines than that in wild type plants at 20 dpi (**Figures 5D,E**). Therefore, these results suggested that *GbEDS1* in *G. barbadense* has a crucial function in response to *V. dahliae* infection. To further investigated the function and potential molecular mechanism of *GbEDS1*,we choose the best resistance transgenic line –L6 as material to carry out the following related experiments.

**Table 2:** Disease grade statistics of *GbEDS1* transgenic and wild type *Arabidopsis.*

Disease level	L5	L6	L10	WT
Level 1	10	11	10	2
Level 2	12	19	18	4
Level 3	4	4	6	15
Level 4	2	1	2	6
Total plants	28	35	36	27
RDI(%)	32.8	31.6	34.0	50.0

### *EDS1* Is Necessary for Conferring Resistance to *Verticillium* Wilt in Cotton

**FIGURE 6:**
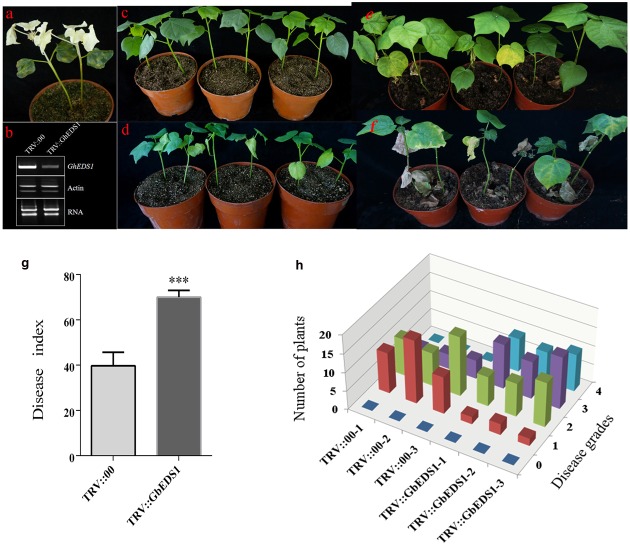
**Silencing of *GbEDS1* resulted in enhancing susceptibility in cotton against *V. dahliae*. (a)** Disease symptoms induced on TRV:00, TRV:GbEDS1 seedlings after inoculation with *V. dahliae*. Ten-day-old *G. barbadense* cv. Pima90-53 seedlings were hand-infiltrated with *Agrobacterium* carrying target gene in the VIGS vector. Two weeks after infiltration, the seedlings were dip-inoculated with *V. dahliae*. Photos were taken at 14 and 28 days post inoculation. **(b)** qPCR analysis of the *GbEDS1* transcripts in control and silencing seedlings. Disease symptoms inoculated with *V. dahliae* strain on TRV::00 **(c,e)** and TRV::GbEDS1 **(d,f)** plants. **(g,h)** The rate of diseased plants and disease index were measured at 20 dpi. Error bars represent the standard deviation of three biological replicates (*n* ≥ 30); asterisks indicate statistically significant differences, as determined by the Student *t*-test (^∗∗∗^*P* < 0.001).

Using VIGS, we monitored the silencing effect with *CLA1*, a gene involved in chloroplast development. Two weeks after *Agrobacterium* infiltration in cotton, silencing of *CLA1* led to an albino phenotype on newly developing true leaves and stem (**Figure 6a**), suggesting that the VIGS approach was successful. Meanwhile, RT-PCR results showed that levels of *EDS1* transcripts were significantly reduced in TRV::GbEDS1 plants when compared with TRV::00 plants (**Figure 6b**). It indicated that *GbEDS1* was effectively silenced in cotton. After inoculation with *V. dahliae* by 14 d, the TRV::00 plants displayed good *Verticillium* wilt resistance (**Figure 6c**) whereas TRV::GbEDS1 seedlings were stunted growth, wilting, chlorosis, and defoliation (**Figure 6d**). With the infection development, the disease symptom became obviously at 28 dpi (**Figures 6e,f**). Values calculated for the rate of diseased plants and DI showed that suppressing the expression of *GbEDS1* in cotton resulted in decreased *V. dahliae* resistance (**Figures 6g,h**). All above results implied that *GbEDS1* played an important role in defense against *V. dahliae*.

### *GbEDS1* Influences *Verticillium* Wilt Resistance by Regulating SA Level

**FIGURE 7:**
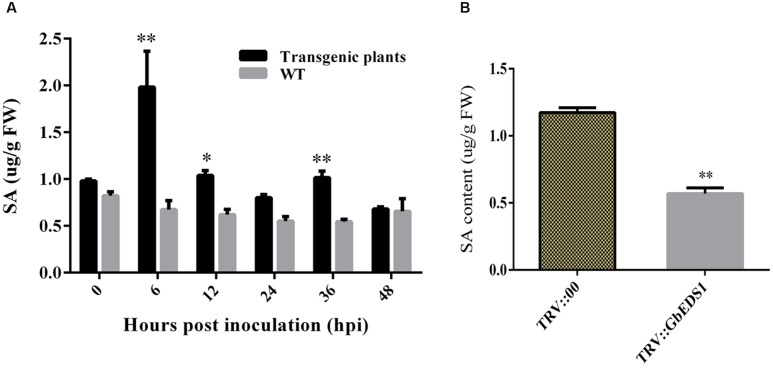
**Detection of SA content in *Arabidopsis* and cotton. (A)** Measurement of SA accumulation in transgenic *Arabidopsis* in response to *V. dahliae* infection. **(B)** Measurement of SA accumulation in silenced cotton. Error bars of SA levels represent the SD of three biological replicates; asterisks indicate statistically significant differences, as determined by the Student’s *t*-test (^∗^*P* < 0.05; ^∗∗^*P* < 0.01).

To learn whether *GbEDS1* regulates the SA signal pathway, we determined the SA levels both in transgenic line and TRV::GbEDS1 plants. After inoculated with *V. dahliae*, the contents of SA were increased both in wild type and transgenic plants. Furthermore, the SA levels in transgenic plants were significantly higher than those in the wild type except for the point at 48 hpi (**Figure 7A**). In contrast, the decreased SA level were detected in *GbEDS1*-silenced plants comparing to the control (**Figure 7B**), indicating that suppressing the expression of *GbEDS1* gene resulted declining SA accumulation in cotton. These results suggested that SA signaling pathway involved in cotton defense against *V. dahliae*, and *GbEDS1* played a positive role in *Verticillium* wilt resistance by regulating the levels of SA.

### *GbEDS1* Overexpression Enhanced the Expression of *NDR1* and *BAK1*

**FIGURE 8:**
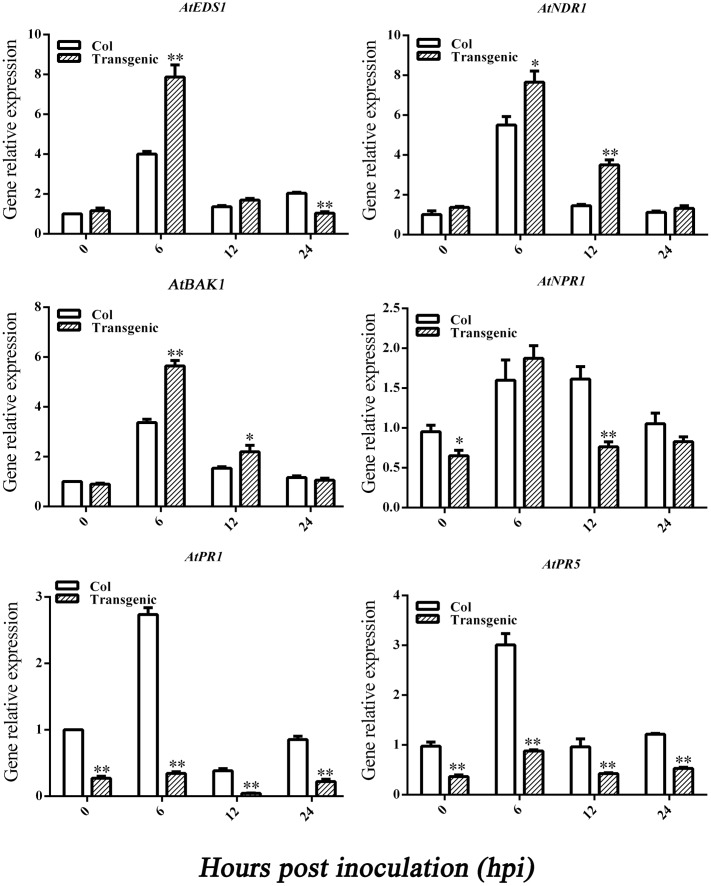
**Detection of SA signaling pathway-related genes and defense related genes.** qRT-PCR analysis the expression of genes (*EDS1*, *NPR1*, *NDR1*, *BAK1*, *PR1*, and *PR5*) in transgenic plants. Error bars represent the standard deviation for three independent experiments, and three technical replicates were analyzed; asterisks indicate statistically significant differences, as determined by the Student’s *t*-test (^∗^*P* < 0.05, ^∗∗^*P* < 0.01).

**FIGURE 9:**
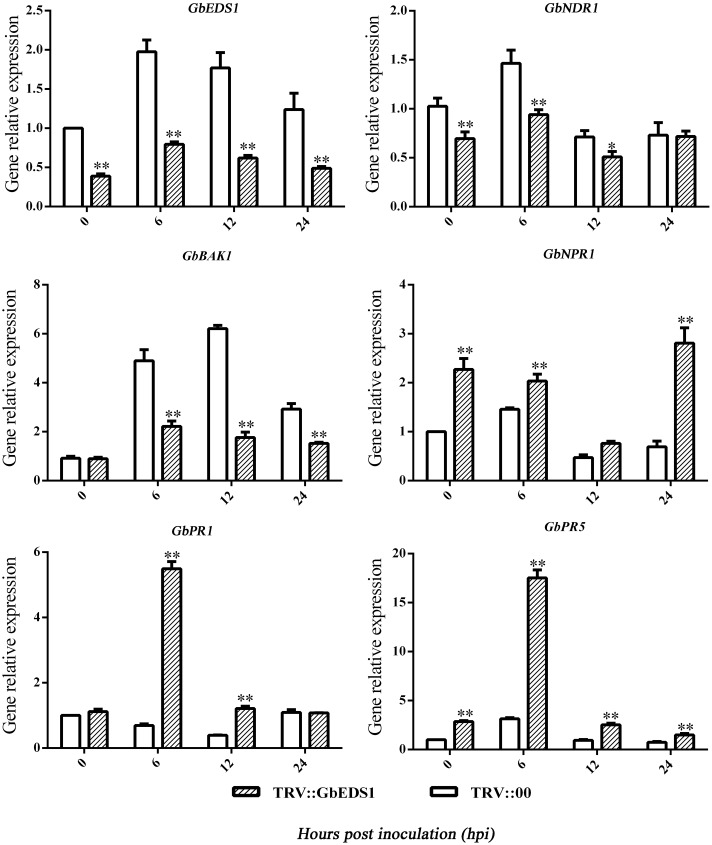
**Detection of SA signaling pathway-related genes and defense related genes.** qRT-PCR analysis the expression of genes (*EDS1*, *NPR1*, *NDR1*, *BAK1*, *PR1*, and *PR5*) in silenced cotton. Error bars represent the standard deviation for three independent experiments, and three technical replicates were analyzed; asterisks indicate statistically significant differences, as determined by the Student’s *t*-test (^∗^*P* < 0.05, ^∗∗^*P* < 0.01).

To learn how *GbEDS1* regulates the SA signal pathway and defense response, we used qPCR to analyze *EDS1*, *NPR1*, *NDR1 BAK1*, *PR1*, and PR5 genes that participate in signal transduction or defense response. As shown in **Figure 8**, the transcripts of *AtEDS1*, *AtNDR1*, and *AtBAK1* were significantly up-regulated in transgenic line than in Col after infection by *V. dahliae*. However, the transcripts of *AtNPR1* and *AtPR1* and *AtPR5* were lower in transgenic lines than that in Col. The opposite trends of above genes expression were obtained in GbEDS1-silenced plants (**Figure 9**). Combination the results from overexpression transgenic line and GbEDS1-silenced plants, it deduced that *GbEDS1* could enhance the expression of *NDR1* and *BAK1* genes, which were reported to mediated the *Verticillium* wilt resistance in cotton (Gao et al., 2011, 2013)

### *GbEDS1* Regulated H_2_O_2_ Content

**FIGURE 10:**
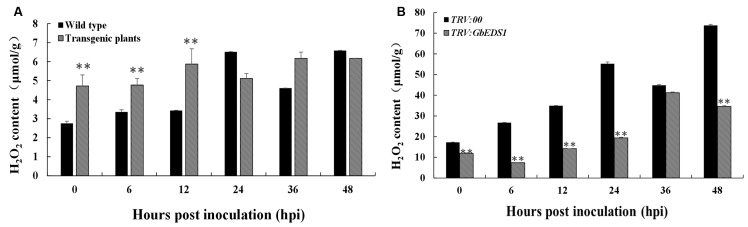
**Measurement of H_2_O_2_ in response to *V. dahliae* infection.** Detection of H_2_O_2_ accumulation in transgenic *Arabidopsis* **(A)** and silenced cotton **(B)**, respectively. Error bars of H_2_O_2_ levels represent the standard deviation of three biological replicates; asterisks indicate statistically significant differences, as determined by the Student’s *t*-test (^∗∗^*P* < 0.01).

**FIGURE 11:**
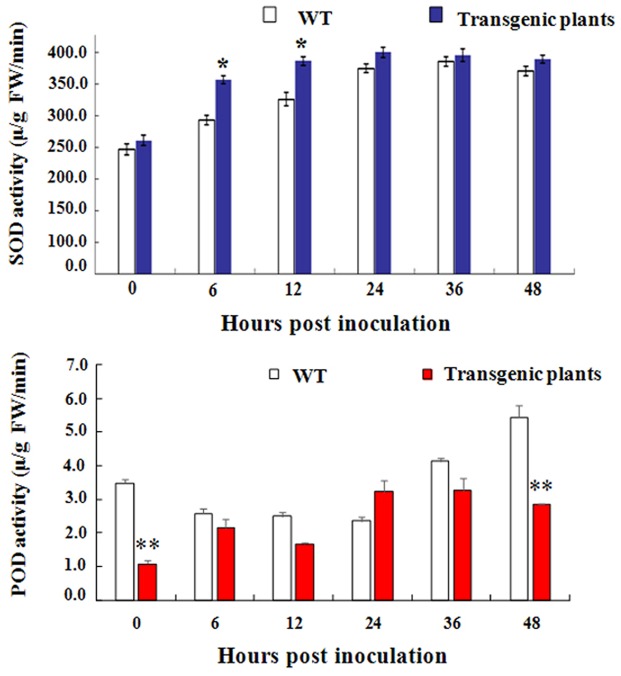
**Detection of SOD and POD activity in transgenic *Arabidopsis*, respectively.** The total activities of the antioxidant enzymes SOD **(A)** and POD **(B)** in *Arabidopsis* plants within 48 h after inoculated with *V. dahliae*. The data are the means ± SD from three independent experiments. The asterisks indicate statistically significant differences between the transgenic and control plants (^∗^*P* < 0.05; ^∗∗^*P* < 0.01, Student’s *t*-test).

H_2_O_2_ accumulation has been considered to be an important parameter in biotic stresses because it usually regarded as an immunity-associated molecule (Camejo et al., 2016). To further investigation the function of *GbEDS1* in defense against *V. dahliae*, we monitored the levels of H_2_O_2_ in transgenic line and GbEDS1-silenced plants. As shown in **Figure 10**, comparing to the wild type, levels of H_2_O_2_ in transgenic plants was higher, especially at 0, 6, and 12 hpi (**Figure 10A**), while the levels of H_2_O_2_ were lower than that in mock plants within 48 h except 36 hpi in GbEDS1-silenced plants (**Figure 10B**). Changes of H_2_O_2_ levels prompted us to assay the major antioxidant enzyme (SOD and POD) activities. According to the results of SOD and POD activities, only several time points exhibited changes comparing to the wild type, however, the whole trend did not obvious, especially in POD activity (**Figure 11**). All above results indicated that overexpression of *GbEDS1* promoted the production of H_2_O_2_ in *Arabidopsis* upon *V. dahliae* infection.

## Discussion

We characterized and analyzed the function of *GbEDS1*, a lipase-like protein gene from *G. barbadense*. The results suggested that *GbEDS1* played a crucial role in *Verticillium* wilt resistance responses by regulating the accumulation of SA, H_2_O_2_. These findings not only broaden our knowledge about the biological role of *GbEDS1*, but also provide new insights into the defense mechanisms of *GbEDS1* against *V. dahliae* in cotton.

Consistent with previous alignments made between GbEDS1 and eukaryotic lipases, its C-terminal region contains a specific motif, KNEDT, which is highly conserved among other *EDS1* genes, e.g., *NbEDS1* and *AtEDS1*. In addition, this motif is an important tool for distinguishing *EDS1* from *PAD4*, another lipase-like gene required for TIR–NB–LRR-type R protein-mediated resistance. Therefore, we propose that *GbEDS1* has biochemical properties and physiological functions similar to those of the previously identified *AtEDS1* and *NbEDS1*. The ORF of *GbEDS1* in this studied is 1,848 bp long and encodes for a protein with 615 aa. These data differ from those previously reported by Su et al. (2014), who cloned a *GbEDS1* gene with a 1647-bp ORF and 548 amino acid residues. According to the sequence analysis of *EDS1* genes from different cotton species, including *GrEDS1*, *GaEDS1*, *GhEDS1*, GbEDS1, indicated that *EDS1* gene is very conservative in cotton. However, the *EDS1* sequence reported by Su et al. (2014) was only about 75% identify with different cotton species reference genomes, and it seems like that this sequence is not a correct sequence of *EDS1* itself in cotton.

To investigate the gene function, we examined the subcellular localization of GbEDS1 protein and found gfp-fluorescence signal both in the nucleus and at the cytomembrane. This is similar to the reported localizations of AtEDS1 and GmEDS1, which were all located to the nucleus and the cytomembrane (Zhu et al., 2011; Wang et al., 2014). Previous study have reported that AtEDS1 forms a complex with PAD4 as well as SAG101, which is preferentially localized in the nucleus and could be translocated to the cytoplasm if an extranuclear form of AtEDS1 existed (Zhu et al., 2011). Our data revealing the nucleus- and cytoplasm-localized GbEDS1 is consistent with the targeting of AtEDS1 in plant cells, also suggesting the functional similarity between the GbEDS1 and AtEDS1.

The genetic basis and molecular mechanisms of *EDS1* and analogous genes have been studied in *Arabidopsis* and other model plant species, including *Lycopersicon esculentum* and *Nicotiana tabacum* (Liu et al., 2002; Hu et al., 2005). However, there was seldom report about *EDS1* in cotton (Su et al., 2014), and how cotton *EDS1* gene function is remain unclear. In this research, we first study the *GbEDS1* function simultaneously by overexpression *Arabidopsis* and silenced cotton. Both the transgenic *Arabidopsis* displaying enhanced resistance and silenced-GbEDS1 cotton showing enhanced susceptibility coincident indicated that *GbEDS1* played a positive role in *Verticillium* wilt resistance. In model plants, *EDS1* regulates the accumulation of SA as part of a positive feedback loop through which it promotes its own expression and the expression of downstream pathway genes (*NPR1* and *PR1*) leading to defense amplification (Feys et al., 2001; Eulgem et al., 2004). In this study, we found that the SA content was higher in transgenic *Arabidopsis* than in wild type, which promoted the expression of *AtEDS1* and *AtBAK1*, however, the expression of *NPR1*, *PR1*, and was not. Previous studies had shown that cotton *NDR1* and *BAK1* mediated the *Verticillium* wilt resistance (Gao et al., 2011, 2013), thus we deduced that *GbEDS1* positively mediated cotton defense through the enhance the expression of *NDR1* and *BAK1*. This special side of *GbEDS1* that differented from other homologous *EDS1* genes was firstly discovered in our study. It was reported that *EDS1* had a‘master’role in coordinating SA and ROS activities in response to abiotic and biotic stress stimuli (Straus et al., 2010). In this study, it proved that *GbEDS1* could regulate SA and H_2_O_2_ content, which were important to defense against *V. dahliae*. Furthermore, the regulating network of *GbEDS1* in coordinating SA and H_2_O_2_ levels remains deeply research.

Although these findings broaden our knowledge about the biological role of *GbEDS1*, further investigation is required. As an important crop plant, our understanding about the molecular biology and functional genomics of cotton lags behind that of other systems, such as *Oryza sativa*, *Triticum aestivum*, and *Zea mays*, largely because molecular tools and resources have, to date, been limited (Gao et al., 2011). It is not as easy to obtain direct genetic evidence and analyze gene functioning because genetic transformation is more difficult with cotton than with other plants. Fortunately, *Agrobacterium*-mediated VIGS can be utilized to silence genes of interest for loss-of-function assays. In addition, unlike more conventional methods for cotton transformation, transient assays based on the VIGS approach are time-saving and are not limited to only a few select cotton cultivars. Therefore, we believe that applying these tools will allow us to obtain a wealth of information about the molecular mechanisms of cotton resistance to fungi, bacteria, nematodes, and viruses. In summary, we characterized GbEDS1, a lipase-like protein from *G. barbadens*e. Expression of *GbEDS1* was up-regulated under *V. dahliae* stress and SA treatment. Overexpression of *GbEDS1* enhanced resistance to *Verticillium* wilt in *Arabidopsis*. Furthermore, silencing of *GbEDS1* in cotton led to enhanced susceptibility to *V. dahliae*. These results indicate that *GbEDS1* plays an important role in the defense response in cotton. Further experiments that obtain stable knockout or overexpression lines in cotton will allow us to elucidate more fully the resistance mechanism of *GbEDS1* in defense against *V. dahliae*.

## Author Contributions

The experiments were designed by WX and MZ, and were conducted by ZY, RW, and YJ. ZY also performed the data analysis and prepared the manuscript along with MZ. All authors approved the final version of the manuscript.

## Conflict of Interest Statement

The authors declare that the research was conducted in the absence of any commercial or financial relationships that could be construed as a potential conflict of interest.

## Footnotes

1 http://www.cbs.dtu.dk/services/SignalP-3.0/

2 http://expasy.hcuge.ch/sprot/prosite.html

3 http://smart.embl-heidelberg.de/

## Abbreviations

DPI: days post-inoculation
*GbEDS1*: *Gossypium barbadense* enhanced disease susceptibility 1
GFP: green fluorescence protein
Hpi: hours post-inoculation
ORF: open reading frame
SA: salicylic acid
VIGS: virus-induced gene silencing
