# Supplementary material for: Island Cotton Enhanced Disease Susceptibility 1 Gene Encoding a Lipase-Like Protein Plays a Crucial Role in Response to Verticillium dahliae by Regulating the SA Level and H2O2 Accumulation
Source: Front Plant Sci. 2016 Dec 15;7:1830. doi: 10.3389/fpls.2016.01830 (PMC5156716; doi:10.3389/fpls.2016.01830)
Supplement: Supplementary file 1 [file Presentation_1.PDF]

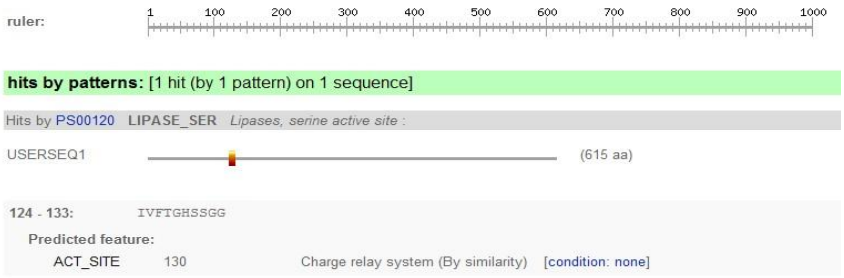

Fig. S1 Analysis of GbEDS1 activity sites using PROSITE program.

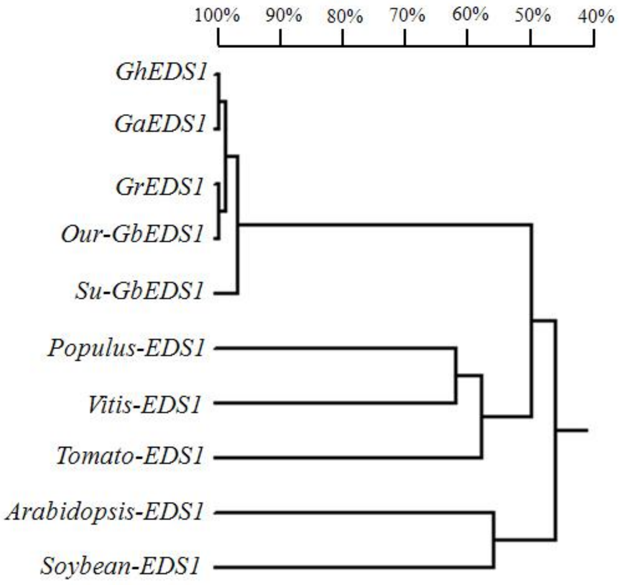

Fig. S2 phylogenetic tree analysis based on the EDS1 complete ORF amino acid sequences from different species. Genebank Accession Numbers for EDS1 proteins: GbEDS1, AY262015; Populus-EDS1, XM-002322106; Tomato-EDS1, ay796114; Soybeam-EDS1, FJ517562; Arabidopsis-EDS1, NM-114678; Vitis-EDS1, EF551159; *GrEDS1*, scaffold251:2115072:2118380; *GaEDS1*, Chr6:9856127:9859250; *GhEDS1*, scaffold821.1:539426:542533.

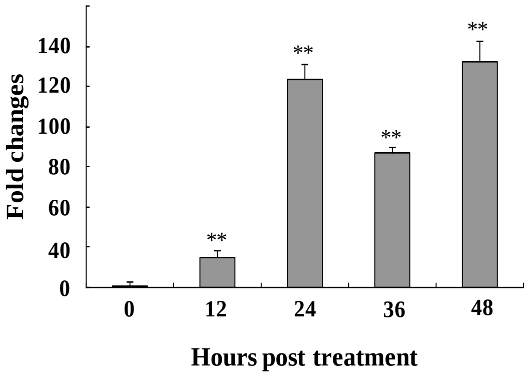

Fig. S3 Expression analysis of *GbEDS1* after induction by MeSA in Pima90-53. Real-time PCR for time-course tests (12, 24, 36, and 48 hpi) of *GbEDS1* expression in response to MeSA. Bars represent levels of *GbEDS1* transcripts relative to those of cotton *GhUBQ14* (for normalization). Seedlings inoculated with water-only served as control. Data are means ± SD of values obtained from triplicate experiments. Asterisks indicate statistical significance (P <0.05, Student's *t*-tests) in comparison with mock control.

Table S1 The primers used in the study

Name	Sequence	Description
GbEDS1-F1	TCTAGAATGGGTAGCCTTACGATTGGGG	ORF
GbEDS1-R1	GAGCTCTTAGATTTCTCTGTTGATCTGCCCCT	ORF
GbEDS1-R2	GAGCTCGATTTCTCTGTTGATCTGCCCCT	Subcellular localization
GhUBQ14-F	CAACGCTCCATCTTGTCCTT	Real-Time PCR
GhUBQ14-R	TGATCGTCTTTCCCGTAAGC	Real-Time PCR
GbEDS1-F3	TCTAGAGTGTTTACTGGACATTCATCTGG	VIGS
GbEDS1-R3	GAGCTCACGGTAGACAAAGAAGCAAG	VIGS
GbEDS1-F4	GTGTTTACTGGACATTCATCTGG	RT-PCR
GbEDS1-R4	CTTGCTTCTTTGTCTACCGT	RT-PCR
GbNDR1-F	CCCGTAACCAAGGAGGCTGT	Real-Time PCR
GbNDR1-R	CTGCTAAGGGAAGGCAAGGATAG	Real-Time PCR
GbNPR1-F	GTCTGGCTGATGTCAATCTGCG	Real-Time PCR
GbNPR1-R	TCCTTCCCTTGCTCTGTCTTGG	Real-Time PCR
GbPR1-F	GGCACAGAACTACGCTAATCAACG	Real-Time PCR
GbPR1-R	GCTTTACCCTCTCACTAACCCACAT	Real-Time PCR
GbPR5-F	GCCAGGGATTCTATCAAACGC	Real-Time PCR
GbPR5-R	ATCCTCGGAGCAATGGGTTC	Real-Time PCR
GbBAK1-F	CGGAATTCGCACACTCGGAGCTGCAAGG	Real-Time PCR
GbBAK1-R	GGGGTACCGAGTGCACAACAGAGCC	Real-Time PCR
ITS1-F	AAAGTTTTAATGGTTCGCTAAGA	Real-Time PCR
ST-VE1-R	CTTGGTCATTTAGAGGAAGTAA	Real-Time PCR
AtRuBisCo-F3	GCAAGTGTTGGGTTCAAAGCTGGTG	Real-Time PCR
AtRuBisCo-R3	CCAGGTTGAGGAGTTACTCGGAATGCTG	Real-Time PCR
AtNPR1-F	CACTATGGCGGTTGAATGTA	Real-Time PCR
AtNPR1-R	GGGAGGAACATCTCTAGGAA	Real-Time PCR
AtPR1a-F	GAAGGCTAACTACAACTACGCTG	Real-Time PCR
AtPR1a-R	TTACACCTCACTTTGGCACAT	Real-Time PCR
AtNDR1-F	TCTTATGGCTTAGTCTCCGTGCG	Real-Time PCR
AtNDR1-R	CGAGGTGGACATCGTCGTAGTAG	Real-Time PCR
AtPR5-F	TCTAAGATGTAACGGCGGCGGAG	Real-Time PCR
AtPR5-R	TGAGGTCAGAGACACAGCCTGCGTA	Real-Time PCR
AtBAK1-F	CAAGCAATCTTTCGGTTGGAGTC	Real-Time PCR
AtBAK1-R	GGCATTAGTATGGCGGTTCACAG	Real-Time PCR
